# Co-circulation of Murray Valley encephalitis virus and Japanese encephalitis virus in south-eastern Australia

**DOI:** 10.1093/jtm/taad059

**Published:** 2023-04-25

**Authors:** Sarah L McGuinness, Colleen L Lau, Karin Leder

**Affiliations:** School of Public Health and Preventive Medicine, Monash University, Melbourne 3004, Australia; Department of Infectious Diseases, The Alfred Hospital, Melbourne 3004, Australia; School of Public Health, The University of Queensland, Brisbane 4006, Australia; School of Public Health and Preventive Medicine, Monash University, Melbourne 3004, Australia; Victorian Infectious Diseases Service, Royal Melbourne Hospital at the Peter Doherty Institute for Infection and Immunity, Melbourne 3000, Australia

## To the editor

We recently published an article on the evolving Japanese encephalitis virus (JEV) situation in Australia in the *Journal of Travel Medicine*.^1^ At the time of writing, 46 human cases of JEV had been notified in Australia since 1 January 2021. However, one previously confirmed case from Victoria has since been reclassified as a confirmed case of Murray Valley encephalitis virus (MVEV) infection, bringing the JEV outbreak total to 45 cases (https://www.health.gov.au/health-alerts/japanese-encephalitis-virus-jev/japanese-encephalitis-virus-jev).

MVEV is a flavivirus endemic to Australia and Papua New Guinea transmitted by *Culex* mosquitoes in an enzootic cycle involving waterbirds.^2^ Major confirmed outbreaks of MVEV were reported across southern and eastern Australia in 1951 (45 cases), 1974 (58 cases) and 2011 (17 cases), with cases in the intervening periods largely confined to Northern Australia.^3^ Five human cases of MVEV infection have been notified in Victoria since December 2022 with three deaths; these are the first confirmed Victorian cases since 1974.^3^^,^^4^ Another four cases of MVEV have been notified in NSW since January 2023, the first confirmed cases in the state since 2011 (https://www.health.nsw.gov.au/news/Pages/20230406_01.aspx). As for JEV, the risk of developing clinical encephalitis following MVEV infection is low (between 1:150 and 1:1000) but associated with case fatality rates of 15–30% and long-term neurological sequelae in 30–50% of survivors.^3^ Clinical features of MVEV overlap with JEV and include fever, headache, seizures (particularly in children) and confusion. There have been at least two documented MVEV cases in international travellers to Australia: one resulted in death and the other in permanent neurological sequelae (partially ventilator-dependent with flaccid quadriparesis).^2^

JEV and MVEV belong to the Japanese encephalitis serogroup of flaviviruses, which also includes another virus endemic to Australia: West Nile virus/Kunjin subtype (WNV/Kunjin).^5^ No human cases of WNV/Kunjin have been reported in Victoria since 2017, but detections in mosquitoes in the Murray–Darling River basin in 2023^4^ indicates co-circulation of JEV, MVEV and WNV/Kunjin with potential risk to residents of and travellers to this area.

The recent emergence of neurotropic flaviviruses in south-eastern Australia has led to new diagnostic recommendations for patients presenting with encephalitis.^5^ Pan-flavivirus serology is recommended for first-line investigation, followed by virus-specific confirmatory testing given cross-reactivity between flaviviruses; serum samples should be collected during the acute illness and at 3–4 weeks after onset.^5^ Molecular detection methods such as PCR on blood, CSF and urine should also be performed for patients presenting early in the course of illness (more likely to be positive in first 7 days).^5^

There are no specific treatment options for JEV, MVEV and WNV/Kunjin. Whilst two JEV vaccines (Imojev® and JEspect®) are available in Australia,^1^ no vaccine is currently available for MVEV or WNV/Kunjin, so education on preventive measures including mosquito bite avoidance is key. Ways to avoid mosquito bites include regular application of an effective insect repellent on exposed skin; wearing long clothing; ensuring accommodation (including tents) is properly fitted with mosquito nets or screens; and using insecticide sprays, vapour dispensing units (indoors) and mosquito coils (outdoors) to repel mosquitoes.

## Funding

National Health and Medical Research Council (NHMRC) Investigator Grants (Grant Numbers 2017229 to S.L.M., 1193826 to C.L.L.); NHMRC Senior Research Fellowship (Grant Number 1155005 to K.L.).

## Author contributions

Sarah McGuinness (Conceptualization-Equal, Data curation-Lead, Writing—original draft-Lead, Writing—review & editing-Equal), Colleen Lau (Conceptualization-Equal, Writing—review & editing-Equal) and Karin Leder (Conceptualization-Equal, Writing—review & editing-Equal).

**Conflict of interest:** None declared.

## Data availability

The data that support the findings of this manuscript are available from the relevant Australian State and Territory Health Department websites (see web links in the text and references).

## Notes

Correspondence letter regarding ‘The evolving Japanese encephalitis situation in Australia and implications for travel medicine’
